# Diseases of the Kidneys

**Published:** 1905-06-17

**Authors:** 


### Progress in Medicine and Surgery.

### DISEASES OF THE KIDNEYS.

(*Continued from page* 189.)

**Essential Albuminuria.**—Posnor[3] has related a case of essential or postural albuminuria during a period of 17 years. The patient, a man, day by day after any bodily exertion, passed albumen in the urine without any symptom of kidney disease. In 1882 he had a slight attack of scarlatina, followed by the appearance of albumen in the urine. He was put to bed and fed on milk, when the albumen promptly disappeared. After the lapse of a week the patient was allowed to get up, and put on a moderate diet; it was then found that the night urine was free from albumen, but that it appeared in the urine as soon as the patient rose from bed. Seventeen years later the same condition existed; the pulse was 70 per minute, the tension of the arteries was normal, there was no trace of dropsy, and the heart was normal. Nothing short of keeping the patient in bed was of any good, and this was obviously out of the question. Moderate diet, abstinence from alcohol, and a certain limitation of bodily exercise, constitute what is efficient as regards treatment.

**Urotropin in Scarlatinal Nephritis.**—Buttersack[4] treated 10 cases of scarlet fever from the commencement of the disease by the administration of urotropin, in doses varying from one to six or seven grains three times a day, according to the age of the patients. In none did nephritis occur. In three severe cases in which slight albuminuria occurred in the third week (the administration of the urotropin having ceased) a repetition of the urotropin treatment caused the albumen to disappear. The author consequently believes that the prophylactic powers of urotropin in scarlatinal nephritis merit further trial.

**Movable Kidney.**—Treves[5] says that it is curious that, considering how easy it is to detect a movable kidney, no mention of the condition is found before the treatise of Pierre Rayer in 1839 on diseases of the kidney. The kidneys are normally maintained in position by the fascia and fat which surround them, by the general pressure of the abdominal viscera, and to a minor degree by the renal vessels. The peritoneum has little effect in holding the gland in place. The kidneys, especially the right, move up and down in respiration, but to a very slight degree. The etiology of movable kidney is obscure; it is much more common in women than in men; it is more frequently met with on the right side, and most cases are discovered between the ages of 25 and 50. A displacable kidney on the right side in a male subject is now and then met with, but such a condition on the left side is exceedingly rare. A large proportion of the women with movable kidney have a feeble muscular development with flabby abdominal walls, and possibly a tendency to general enteroptosis. The trouble is more common in those who have had many children than in those who are childless, and quite a striking proportion of the subjects have become more or less rapidly thin. On the other hand, this state of the organ is by no means of necessity a feature of emaciation. The mobility of such kidneys is very variable—in the slighter varieties the lower end can be felt but not held, in the most advanced the kidney is floating, and can be felt during expiration by mere palpation. The movable kidney may become the seat of hydronephrosis, due to the repeated kinking or bending of the ureter whereby the escape of urine is abruptly hindered. This is marked by acute symptoms. The most common conditions simulating movable kidney are Reidel's lobe, a distended gall bladder, and a fæcal mass in the colon. The classical symptoms of the disorder are a sense of dragging in the loin or abdomen, with an undefinable discomfort and feeling of weakness which may pass into actual pain, which may follow the lines accredited to renal pain or radiate down the thighs and legs and across the back. Dyspepsia, flatulence, and constipation may also occur. The symptoms are increased by movements, whilst the recumbent position usually gives complete relief. Among the less common effects of movable kidney are dilatation of the stomach, intestinal obstruction, and jaundice. Treatment by operation is imperative in cases where there have been "torsion symptoms"; in practically all other cases nephrorhaphy is quite uncalled for. In the first place, the operation, though unattended by any mortality, is very often (20 per cent.) unsuccessful; and, secondly, a cessation of the symptoms can be obtained by less severe measures. Suturing the kidney will become one of the rare operations of surgery. The treatment should consist of rest in the recumbent position, careful feeding, attention to the digestive organs, and massage, for a month. This rids the patient of her symptoms, and especially of the often accompanying neurasthenia. At the end of this period a properly fitting truss should be carefully fitted. At Treves' request Mr. Ernst contrived a truss which imitates the pressure of the hand and keeps the kidney in its proper position. In more than 95 per cent. of upwards of 300 cases the truss proved absolutely efficient, the kidney has been kept in place, and the distress that had existed has entirely vanished. In a large proportion of cases the truss can be given up at the end of 18 months or two years.

**Movable Kidney.**—Moullin[6] has recorded some cases of movable kidney in which the symptoms simulated gastric ulcer, biliary colic, and appendicitis. The first case was that of a woman, aged 44, who complained of pain in the epigastrium coming on half an hour after meals. Solid food made it worse, vomiting was frequent and was rather encouraged, as it relieved the pain. Scarcely a day passed without at least one attack. This had lasted some 20 years. Twenty-one months before there had been three attacks of hæmatemesis, and there was melæna at the same time. The stomach was not dilated or misplaced, and there was a little tenderness in the epigastrium, but no tumour. Both kidneys were movable, especially the right one, which descended so far that the hands could be made to meet above it. Whilst lying in bed the vomiting ceased and the pain after food became very much less. The right kidney was sutured in place, with the result that the pain and vomiting were completely cured. In two cases there were repeated attacks of colic beginning in the right hypochondrium, with, in one, attacks of jaundice lasting two or three days. The liver was not enlarged and no tumour in the region of the gall-bladder could be felt; and in both rest in bed caused cessation of the attacks. In both the lower end of the right kidney could easily be felt when the patients were in the upright position. These two cases were due, not to a slipping up and down of the kidney behind the peritoneum, but to a tilting forward of the upper end, so as to assume almost a horizontal position. Suture of the kidney so that it could not move cured the patients. In another case of movable kidney there were recurrent attacks of colic with constipation and abdominal tenderness brought on by exercise or trivial indiscretion in diet, simulating appendicitis. The appendix was found to be healthy and was not removed, whilst nephrorhaphy cured.

[3] Med. Chron., January. [4] Ibid. [5] Pract., January. [6] Lancet, Dec. 10.

